# Supplementary material for: Splice-Junction-Based Mapping of Alternative Isoforms in the Human Proteome
Source: Cell Rep. Author manuscript; Available in PMC 2020 Jan 15. (PMC6961840; doi:10.1016/j.celrep.2019.11.026)
Supplement: 3 [file NIHMS1546469-supplement-3.zip › DF2/PXD009737/TestisTrypsin-9-Q9UNF0-ATDGVTLTGINQTGDQSLPSKPSSVSSYEK.pdf]

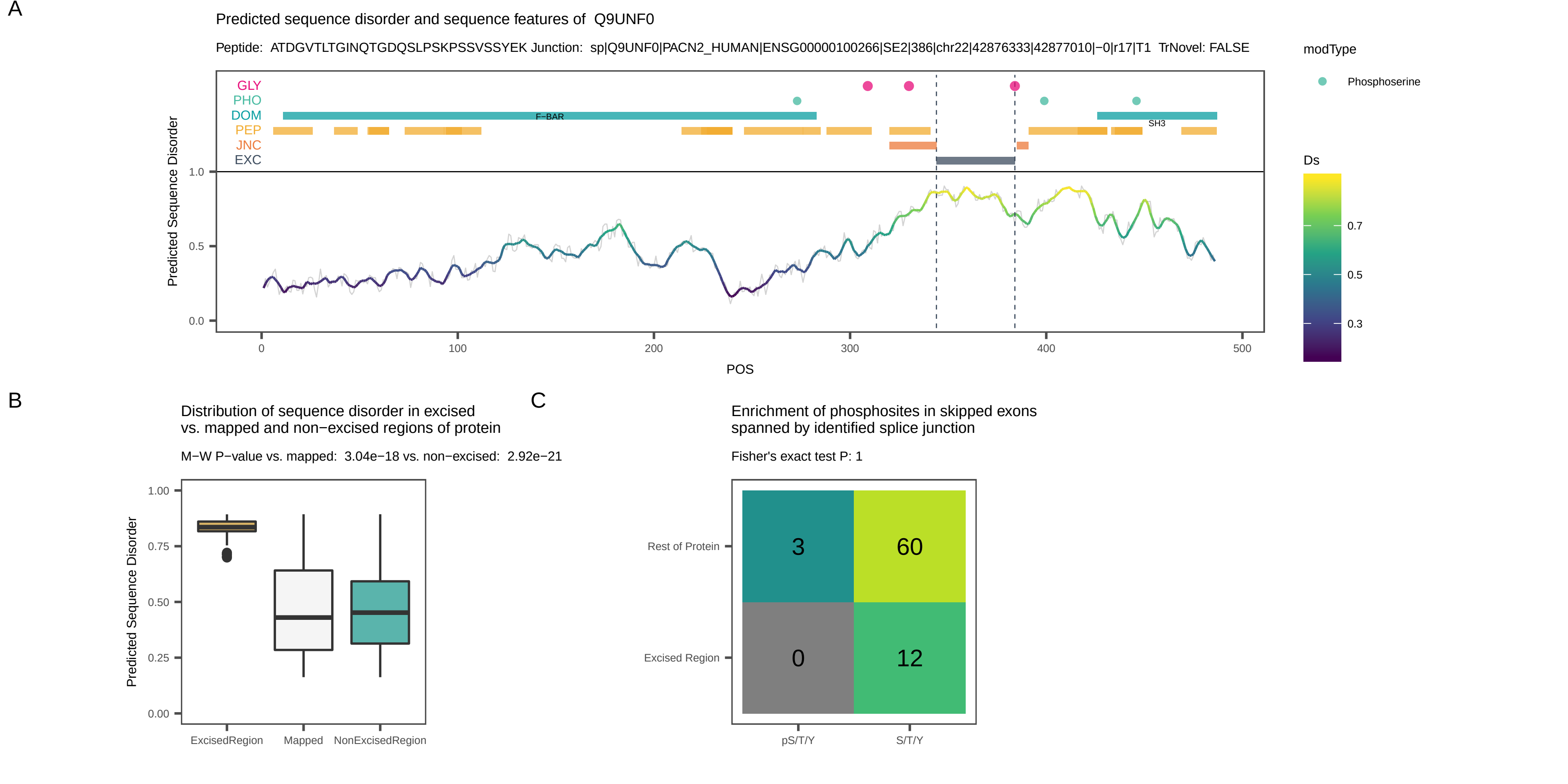

A
Predicted sequence disorder and sequence features of Q9UNF0
Peptide: ATDGVTLTGINQTGDQSLPSKPSSVSSYEK Junction: sp|Q9UNF0|PACN2_HUMAN|ENSG00000100266|SE2|386|chr22|42876333|42877010|−0|r17|T1 TrNovel: FALSE
GLY
PHO
DOM
PEP
JNC
EXC
F−BAR
SH3
Predicted Sequence Disorder
POS
modType
Phosphoserine
Ds
B
Distribution of sequence disorder in excised vs. mapped and non−excised regions of protein
M−W P−value vs. mapped: 3.04e−18 vs. non−excised: 2.92e−21
Predicted Sequence Disorder
ExcisedRegion
Mapped
NonExcisedRegion
C
Enrichment of phosphosites in skipped exons spanned by identified splice junction
Fisher's exact test P: 1
Rest of Protein
Excised Region
3
60
0
12
pS/T/Y
S/T/Y
